# Multiple Sclerosis Lesion Detection Using Constrained GMM and Curve Evolution

**DOI:** 10.1155/2009/715124

**Published:** 2009-09-10

**Authors:** Oren Freifeld, Hayit Greenspan, Jacob Goldberger

**Affiliations:** ^1^Department of Biomedical Engineering, Tel-Aviv University, Tel Aviv 69978, Israel; ^2^School of Engineering, Bar-Ilan University, Ramat-Gan 52900, Israel

## Abstract

This paper focuses on the detection and segmentation of Multiple Sclerosis (MS) lesions in magnetic resonance (MRI) brain images. To capture the complex tissue spatial layout, a probabilistic model termed Constrained Gaussian Mixture Model (CGMM) is proposed based on a mixture of multiple spatially oriented Gaussians per tissue. The intensity of a tissue is considered a global parameter and is constrained, by a parameter-tying scheme, to be the same value for the entire set of Gaussians that are related to the same tissue. MS lesions are identified as outlier Gaussian components and are grouped to form a new class in addition to the healthy tissue classes. A probability-based curve evolution technique is used to refine the delineation of lesion boundaries. The proposed CGMM-CE algorithm is used to segment 3D MRI brain images with an arbitrary number of channels. The CGMM-CE algorithm is automated and does not require an atlas for initialization or parameter learning. Experimental results on both standard brain MRI simulation data and real data indicate that the proposed method outperforms previously suggested approaches, especially for highly noisy data.

## 1. Introduction

 Multiple sclerosis (MS) is the most common nontraumatic neurological disease in young adults. It is an inflammatory demyelinating disease that is primarily associated with axonal loss and formation of lesions in the central nervous system [1]. Magnetic Resonance Imaging (MRI) is the leading diagnostic tool in the context of MS. The extensive use of this imaging technique has significantly improved our understanding of the disease [2]. MRI is currently being used for MS diagnosis, assessment of disease progression, and evaluation of the efficiency of drug therapy [3]. The most common quantitative parameter is the *lesion burden* (or *load*) of the disease expressed in terms of the number and volume of the brain lesions. The MRI measured lesion burden is correlated with clinical findings [4–8]. However, it should be stated that the exact relationship between MRI measured lesions and pathological findings is not entirely clear [9–11]. MRI is also capable of providing measures and visualization of subclinical activity. Last but not least, it is sensitive to temporal variations that are typical to this disease [12]. 

A correct segmentation of healthy tissues is crucial for successful detection and segmentation of MS lesions [13]. Moreover, one of the characteristics of the disease is a decrease in the volume of healthy tissues, especially gray matter (GM). Having said that, it is clear why, in spite of the fact that the present work focuses on MS lesion detection and segmentation, the topic of healthy tissue segmentation should be addressed as well.

MRI brain image segmentation is a challenging task, even with healthy patients. The challenge stems from several reasons. First, MRI images present various noise artifacts such as intratissue noise, partial-volume effects and thermal noise [14]. These artifacts may cause unrealistic results, where tissue regions as well as lesion regions may appear granular, fragmented, or violating anatomical constraints. Second, the geometry of the brain tissues is complex and cannot be modeled by simple geometric primitives. Third, there is a significant variability in tissue geometry between patients. Finally, intensity distributions of different tissues can present a notable overlap. Segmentation of abnormal structures like MS lesions, is a difficult task. Lesions differ from each other in size, location and shape. Also, as each MRI modality reflects different physical properties, the exact lesion boundaries may vary between different MRI channels. To complicate matters, the number of voxels that are associated with MS lesions is significantly smaller than the number of voxels that are associated with healthy tissues. Thus, simple clustering algorithms (e.g., K-means), fail to discern MS-lesion voxels from the rest of the image.

With notable exceptions (e.g., [15, 16]), segmentation of MRI brain images of MS patients is usually addressed by probabilistic voxel-based intensity modeling. Some of the salient aspects of these works are described below. 

### 1.1. Parametric and Nonparametric Modeling

Several authors suggested to model the intensity of the brain tissues using a mixture of Gaussian probability density functions [17–22]. In this model, heron termed *Gaussian Mixture Model* (GMM), the intensity of a voxel is assumed to be drawn from the specific normal distribution associated with its parenting tissue. The GMM belongs to the category of parametric classification methods, since it is fully defined by its parameter set. The present work also belongs to this category. Other researchers have suggested using nonparametric methods such as the K-Nearest Neighbor (KNN) classification rule, or Parzen windows [23, 24]. These methods avoid making any assumption on the probability density function that is underlying the data. Instead, they try to locally estimate the probability density function, based on some labeled prototypes. These prototypes are given manually by a human expert, so such methods are not completely automated. A combination of parametric and nonparametric methods has been suggested recently [13]. 

### 1.2. MS Lesions: Explicit Model or Outliers?

While several authors (e.g., [13, 23]) model the MS lesions as a distinguished class in addition to the healthy tissue classes (CSF, GM, WM), another approach is to model the lesions as outliers. For example, Van Leemput et al. [19] treat voxels that are not well explained (by either of the healthy tissue models) as candidates to be classified as lesions. Among these candidates, the separation into lesion and nonlesion voxels is made according to contextual information and a set of rules. It is also possible to treat lesions as outliers only in an intermediate stage, and then to build an explicit model for them [22]. 

### 1.3. Incorporating Spatial Information

A segmentation method that relies solely on the voxel intensity is unlikely to produce sufficient results. As such, many efforts have been made to incorporate spatial information within the segmentation process. One approach is the use of a statistical atlas [17–19, 25]. The atlas provides an a-priori probability regarding expected tissue location. A similar approach is the template-driven segmentation [20, 23, 24] that employs a deformable digital anatomical atlas to extract white matter masks using nonlinear registration. The main purpose is to reduce the number of misclassifications, by assuming that lesions are usually placed within the WM tissue. Atlas/template based approaches make a powerful tool for image segmentation; however, they suffer from several disadvantages. They require registration of the atlas/template to the brain target. Image registration is a computationally intensive procedure, and not always applicable. Also, registration errors usually result in segmentation errors. Registration is especially problematic when the patient presents significant anatomical variability with respect to the atlas/template, which is often the case in MS patients. Another conventional method to improve segmentation smoothness and immunity to noise is to use a Hidden Markov Random Field (HMRF), thus modeling neighboring voxel interactions [13, 18, 19]. Smoother structures are obtained in the presence of moderate noise as long as the HMRF parameters controlling the strength of the spatial interactions are properly selected. HMRF based algorithms are computationally intractable unless some approximation is used which still requires computationally intensive algorithms.

In very recent works new approaches are starting to emerge. In [15] regional properties are used in a multifeature and multiscale approach combining segmentation and classification (supervised) for MS lesion analysis. In [26] a 4D space (*T*_1_ intensity and spatial features) multiGaussian model is used to model each tissue. A large number of Gaussians is used per brain tissue to capture the complex spatial layout. The intensity of a tissue is considered a global feature and is incorporated into the model by parameter tying of all related Gaussians. The model, termed Constrained GMM (CGMM), was shown in [26] to provide accurate segmentation of *T*_1_ simulated and real MRI brain images into the three healthy tissues, in particular in noisy and low-contrast MRI images, without the need for coregistration of the input image and an atlas. In the current work we propose an extension to the CGMM model that can handle multisequence MRI data with a focus on MS lesion detection and segmentation. We also suggest a new and efficient way for approximating the CGMM computations. Another focus of this work is utilizing the CGMM as a good initialization for a level set method that provides a refined lesion boundary. We show that direct implementation of curve evolution methods to the complicated MRI image provides poor brain segmentation and no detection of MS lesions. The lesion detection obtained using the CGMM makes it possible to apply active contour techniques for lesion detection. A preliminary version of this paper appeared in [27].

The paper is organized as follows. Section 2 presents the CGMM framework. Lesion detection based on the CGMM and lesion boundary refinement via a level-set method, hereon termed CGMM-CE, are described in Sections 3 and 4, respectively. Algorithm validation on both simulated and real brain volumes is shown in Section 5. Discussion and conclusions are presented in Section 6.

## 2. The CGMM Segmentation Framework

The complex spatial layout of an MRI brain image poses a challenge for conventional intensity based GMM modeling schemes. To accommodate the spatial complexity, we model an image as if its voxels were drawn independently from a mixture of * many* Gaussians:

(1) $$\begin{array}{c} f\left(x,I\left(x\right)\right)=\overset{n}{\underset{i=1}{\sum }}\alpha_{i}f_{i}\left(x,I\left(x\right)\;|\;\mu_{i},\Sigma_{i}\right) \end{array}$$

such that *x* is the 3D position information included in the feature vector, *I*(*x*) is the intensity vector associated with the voxel located at position *x*, *n* is the number of components in the mixture model, *μ*_*i*_ and Σ_*i*_ are the mean and the covariance of the *i*th Gaussian component *f*_*i*_, and *α*_*i*_ is the *i*th mixture coefficient. Considering the location of the voxel as an additional feature enables us to incorporate spatial information into the probabilistic model. Each Gaussian in the GMM provides a probabilistic model for a specific small area in the MRI image.

The spatial shape of the tissues is highly nonconvex. However, since we use a mixture of many components, each Gaussian component models a small local convex region. The intravariability of the intensity features within a tissue is significantly less than the intervariability among different tissues. It is therefore sufficient to model the intensity variability within a tissue by a single Gaussian (in the intensity features). To incorporate this insight into the model, each Gaussian is linked to a single tissue and all the Gaussians related to the same tissue share the same intensity parameters. We also assume that locally, the intensity and spatial features are uncorrelated. The above assumptions impose the following structure on the mean and covariance of the Gaussian components:

(2) $$\begin{array}{c} \mu_{i}=\left(\begin{array}{c} \mu_{i}^{x}\\ \mu_{\pi (i)}^{I} \end{array}\right),\;\;\Sigma_{i}=\left(\begin{array}{cc} \Sigma_{i}^{x} & 0\\ 0 & \Sigma_{\pi (i)}^{I} \end{array}\right), \end{array}$$

where *x* is the spatial feature vector, *I* is the intensity feature vector, *π*(*i*) is the tissue that is linked to the *i*th Gaussian component, *μ*_*i*_^*x*^ and Σ_*i*_^*x*^ are the spatial mean and covariance parameters of the *i*th Gaussian component, and *μ*_*j*_^*I*^ and Σ_*j*_^*I*^ are the intensity mean and covariance parameters shared by all the Gaussian components that are linked to the *j*th tissue. The Gaussian component *f*_*i*_ has, therefore, the following form:

(3) $$\begin{array}{c} f_{i}\left(x,I\left(x\right)\;|\;\mu_{i},\Sigma_{i}\right)=𝒩\left(x;\mu_{i}^{x},\Sigma_{i}^{x}\right)\times 𝒩\left(I\left(x\right);\mu_{\pi \left(i\right)}^{I},\Sigma_{\pi \left(i\right)}^{I}\right). \end{array}$$

**Figure 1:**
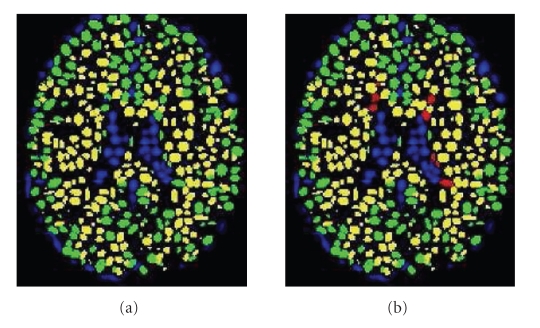
Illustration of the GMM representation. (a) Segmenting the brain image into three main tissues. (b) Adding a fourth class for MS lesions; Color Legend: Blue-CSF, Green-GM; Yellow-WM; Red-MS.

The main advantage of the CGMM framework is the ability to combine, in a tractable way, a local description of the spatial layout of a tissue with a global description of the tissue's intensity. The multiGaussian spatial model makes our approach much more robust to noise than intensity-based methods. Note that no prior atlas information is used in the modeling process. An illustration of the CGMM model applied to the three tissues (CSF, GM, and WM) is shown in Figure 1(a). In case of MS lesion segmentation we consider the lesion matter in the CGMM modeling as a fourth tissue in addition to the three healthy tissues (Figure 1(b)).

### 2.1. Parameter Estimation via the EM Algorithm

Given an MRI image with *T* voxels: {(*x*_*t*_, *I*(*x*_*t*_)) | *t* = 1,…, *T*}, the likelihood of a given CGMM parameter set is

(4) $$\overset{T}{\underset{t=1}{\prod }}\;\overset{n}{\underset{i=1}{\sum }}\alpha_{i}f_{i}\left(x_{t},I\left(x_{t}\right)\;|\;\mu_{i},\Sigma_{i}\right).$$

Initially, the model parameters are, of course, unknown. To find the maximum-likelihood parameters we utilize the classical Expectation-Maximization (EM) algorithm [28]. The EM algorithm handles the parameter estimation task by iterating the E and M steps. In the E-step, it treats the parameter set as given, and estimates the probabilities of each sample to be drawn from each Gaussian. Informally, this step can be seen as soft segmentation (but here, the number of segments is *n*, the number of Gaussians, and not *K*, the number of the tissues). The M-step performs parameter reestimation. Thus, the equations take the following form.

**E-Step:**

(5) $$\begin{array}{ll} w_{it} & =p\left(i\;|\;x_{t},I\left(x_{t}\right)\right)\\ & =\frac{\alpha_{i}f_{i}\left(x_{t},I\left(x_{t}\right)\;|\;\mu_{i},\Sigma_{i}\right)}{\sum_{j=1}^{n}\alpha_{j}f_{j}\left(x_{t},I\left(x_{t}\right)\;|\;\mu_{j},\Sigma_{j}\right)}\;i=1,\ldots ,n,\;\;t=1,\ldots ,T \end{array}$$

*w*_*it*_ can be viewed as the posterior probability that voxel *t* was created using Gaussian *i*.

**M-Step**

Let

(6) $$\begin{array}{ll} n_{i} & =\overset{T}{\underset{t=1}{\sum }}w_{it}\;i=1,\ldots ,n,\\ k_{j} & =\underset{i|\pi (i)=j}{\sum }n_{i}\;j=1,\ldots ,k \end{array}$$

with *n*_*i*_ and *k*_*j*_ interpreted as the expected number of voxels associated with the *i*th Gaussian and *k*th tissue, respectively, and *T* is the number of voxels in the MRI image. Parameter reestimation is then defined as follows.

Spatial parameters:

(7) $$\begin{array}{ll} \mu_{i}^{x} & =\frac{1}{n_{i}}\overset{T}{\underset{t=1}{\sum }}w_{it}x_{t},\\ \Sigma_{i}^{x} & =\frac{1}{n_{i}}\overset{T}{\underset{t=1}{\sum }}w_{it}\left(x_{t}-\mu_{i}^{x}\right){\left(x_{t}-\mu_{i}^{x}\right)}^{⊤},\\ \alpha_{i} & =\frac{n_{i}}{T}. \end{array}$$

Intensity parameters:

(8) $$\begin{array}{ll} \mu_{j}^{I} & =\frac{1}{k_{j}}\underset{i|\pi (i)=j}{\sum }‍\;\overset{T}{\underset{t=1}{\sum }}w_{it}I\left(x_{t}\right),\\ \Sigma_{j}^{I} & =\frac{1}{k_{j}}\underset{i|\pi (i)=j}{\sum }‍\;\overset{T}{\underset{t=1}{\sum }}w_{it}\left(I\left(x_{t}\right)-\mu_{j}^{I}\right){\left(I\left(x_{t}\right)-\mu_{j}^{I}\right)}^{⊤}. \end{array}$$

Note that while the spatial parameters are estimated separately for each Gaussian, the estimated intensity parameters are the same for all the Gaussians that belong to the same tissue. The grouping function *π* that links between the Gaussian components and the tissues is learned in the initialization step and is not altered by the EM iterations. Therefore, the affiliation of a Gaussian component to a tissue remains unchanged. However, since the learning is performed simultaneously on all the tissues, voxels can move between tissues during the iterations.

Once the optimal set of parameters is obtained using the EM algorithm, we can compute soft tissue segmentation maps. Moving from soft segmentation to hard segmentation is straight forward, using the Maximum-A-Posteriori (MAP) criterion. Tissue segmentation is achieved by the affiliation of each voxel to the tissue that maximizes the a posteriori probability:

(9) $$\begin{array}{ll} \text{tissue-label}\left(\text{voxe}{\text{l}}_{t}\right) & =\underset{j}{\arg\;\max\;}\;\;p\left(\text{tissue}=j\;|\;x_{t},I\left(x_{t}\right)\right)\\ & =\underset{j}{\arg\;\max\;\;}\;\underset{i\;|\;\pi (i)=j}{\sum }\alpha_{i}f_{i}\left(x_{t},I\left(x_{t}\right)\;|\;\mu_{i},\Sigma_{i}\right). \end{array}$$

**Figure 2:**
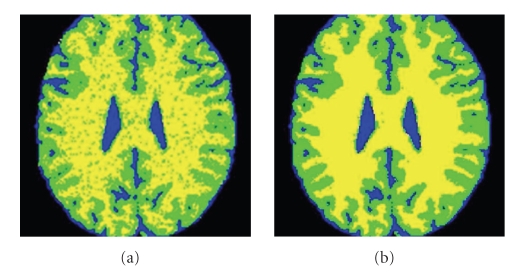
Illustration of the influence of the EM iterations. As the parameter estimation improves with the iterations, the segmentation induced from the model becomes more and more robust to noise. Blue-CSF; Green-GM; Yellow-WM. (a) Initial segmentation. (b) Segmentation after convergence (five EM iterations).

Figure 2 illustrates the segmentation induced from the CGMM model and shows how the EM iterations improve the segmentation quality. Since a lot of the algorithmic effort is spent on finding a good initialization (see Section 2.2), the EM needs only few iterations to converge.

### 2.2. Model Initialization

In Section 2.1, we saw how the EM algorithm is applied to the MRI data to find the maximum-likelihood parameter-set for the CGMM. However, the EM is notoriously known to get stuck in local maxima. Hence, appropriate initialization is required. In the first step the K-means clustering is done based only on the intensity features (*T*_1_, *T*_2_, and *PD*), which gives a good initial segmentation into the three major tissue classes. We utilize *T*_1_ to give tissue labels to the three groups.

Given the intensity K-means clustering of the voxels into the three tissues, we want to model each tissue with many small locally convex Gaussians. it is important that small Gaussians will be allowed, as lesions are often very small, and we would like the Gaussians layout to capture them. We have found empirically that randomly selecting 1/20 of the voxels as initial Gaussian centers yields a resolution that is high enough for detecting the MS lesions. To ensure that small isolated areas are explicitly represented by local Gaussians, we first apply a three-dimensional connected component process to each tissue. If a connected component contained less than three voxels, it was ignored and disregarded as noise. For each connected component (of voxels all from the same tissue) a subset of voxels is randomly chosen and a new Gaussian is formed at each of the chosen voxels. To compute initial covariance values for each Gaussian we assign each voxel to the nearest center with the same tissue label. The Gaussian covariance (both intensity and spatial) is computed based on all the voxels that are assigned to the Gaussian center. As a result of the initialization process each Gaussian is linked to one of the tissues and each voxel is affiliated with a selected Gaussian. 

### 2.3. Efficient EM Based on the KDT Transform

The strength of the proposed model relies on the highly detailed spatial representation obtained by using many small Gaussians such that each Gaussian represents a small coherent and convex region. Of course, the number of Gaussians that are required to cover the entire image increases as the size of the Gaussians decreases. The main implementation problem is the large number of Gaussians, that leads to high computational complexity of the EM algorithm. The computational complexity involved with the E-step is *O*(*nT*) where *T* is the number of voxels and *n* is the number of Gaussian components. A typical number of voxels in 3D MRI brain images is on the order of 10^6^. The number of Gaussians in the CGMM framework may be as high as 10^3^ or even 10^4^ when segmentation of MS lesions is also required. This fact hinders the E-step computations significantly.

It is clear that voxels that are very far away from the spatial mean of a particular Gaussian *i*, have a small chance to originate from this Gaussian. Due to numerical limitations, the probability density of each Gaussian vanishes whereas the actual distance from the spatial mean is still finite. Thus, for practical purposes, there is no need to compute in the E-step the weights *w*_*it*_ for every Gaussian *i* and every voxel *t*. Instead, for every voxel we maintain a small list of its nearest Gaussians. Our experiments showed that even a small number of Gaussian neighbors (say 10) gives rise to very accurate approximations of the model and does not cause any degradation in the segmentation performance. The computational complexity is *O*(10*T*) and therefore using this procedure, the number of computations in each E-step is reduced by a few orders of magnitude.

We note in passing that this approach is superior to maintaining a list of voxels for every Gaussian. The reason is that in the latter, we may find that there are voxels that are explained by no Gaussian at all. Admittedly, maintaining a list of nearest Gaussians for every voxel may result in Gaussians that have, if any, very little voxels. However, this should not compromise the results, since such Gaussians will simply shrink and die off during the EM process.

The main problem is efficiently finding the list of nearest Gaussians for each voxel. In this section we suggest an efficient way to find this list. The method is based on the K-Distance Transform (KDT) [29], (and can be applicable to other GMM applications defined on a grid in which the number of Gaussians is large). KDT, introduced by Warfield [29] and later improved by Cuisenaire [30], is an extension to the Distance Transform (DT). Originally, the KDT was used for fast implementation of the kNN classification rule. The KDT input is a discrete Euclidean space containing prototypes and nonprototypes. Each prototype is given a unique identifier. The output is a set of identifier maps. The first map is a first Nearest-Neighbor (NN) map. In effect, each element in this map contains the identifier of the prototype that is the nearest to this element. The second map is a second NN map and so on. The brute force approach for KDT computation is very simple and extremely inefficient: for every space element, compute its distances from all the given prototypes, sort them by the distances, and select only the nearest neighbors. Clearly, this approach is not applicable to large datasets due to its high complexity. Warfield proposed an efficient way for fast KDT computation, that is based on local propagations, rather than direct computations. The main point is that local propagations, implemented using several raster scans, require a number of computations that is only linear in the number of elements in the discrete space [29].

In our work, the input prototypes can be taken as the spatial means of the Gaussians and applying the KDT algorithm, we obtain for every voxel a list of its nearest Gaussians. We can use the Mahalanobis distance as the metric of choice. Note that the Mahalanobis distance depends not only on the locations of the prototype (in effect, the spatial mean of the Gaussian) and the voxel, but also on the covariance matrix of the Gaussian. Experiments indicate that while EM iterations modify the spatial parameters to some extent, they are unlikely to cause a Gaussian to wander far away from its original vicinity. Thus, it is enough to apply the KDT for computing the list of nearest Gaussians only once. There is in fact a chance that the list of the nearest Gaussians will change slightly, but the Gaussians that will be replaced are likely to be far from the voxel so that their contribution is not important. Here too, our experiments indicate that any discrepancy between the approximated model and the true model is intangible.

Note that in the present work, the importance of the KDT is primarily in approximating the exact computation of the probabilistic model. However, it also gives us contextual information. Not only is it possible to know which are the *K* nearest Gaussians for every voxel, but also their tissue affiliation. Hence, the KDT immediately gives additional useful information: for every voxel it is possible to know, which tissue its nearest Gaussians relate to. Such knowledge can be beneficial to determine if a voxel is located within a WM area and is used in the following section to obtain a fast implementation of rules for detecting MS lesions.

## 3. Lesion Detection Utilizing the CGMM

A major focus of the current work is to extend the CGMM framework to MS lesion segmentation. Section 2.2 described that one of the very first initialization steps was intensity-based K-means clustering. As mentioned earlier, simple clustering methods for MS lesion detection are usually inapplicable, due to the small number of lesion voxels. We present a novel approach to MS lesion detection, that exploits the Gaussians layout of the CGMM. First, the model is initialized with three tissue classes, and its parameters are learned. Due to the fact that MS lesion voxels are significantly outnumbered by the healthy tissue voxels, the clusters still succeed in representing the three healthy tissues (CSF, GM, and WM). The lesion voxels are of course misclassified at this point as one or more of the healthy tissues. Moreover, at this stage there are *misclassified Gaussians*, that is, Gaussians labeled as healthy tissues that are supposed to be labeled as MS. The purpose of the current stage is to identify these Gaussians, and change their labels accordingly. A major distinction of the present work from previous works is that *MS lesion detection is performed on the Gaussian level rather than on the voxel level*.

For each Gaussian a decision is made, based on its features, whether it should in fact be labeled as MS, or remain labeled as one of the healthy tissues. Both supervised and unsupervised approaches can be used to deal with this task. For example, a rule based system can be used to distinguish the MS lesion Gaussians from the normal tissue Gaussians. A Gaussian for which all these conditions hold, is labeled as MS lesion. An example of such a rule set for Gaussians labeled as GM is the following:

1. *T*_2_ mean-intensity of the Gaussian > *T*_2_ mean-intensity of the GM tissue + *ϵ*_GM,*T*_2__,
2. *T*_2_ mean-intensity of the Gaussian > *PD* mean-intensity of the GM tissue + *ϵ*_GM,*PD*_,
3. a large Mahalanobis distance between the mean-intensity of the Gaussian and the mean-intensity of the CSF/GM/WM tissue,
4. the majority of Gaussian's *K* nearest Gaussians are labeled as WM,

where *ϵ*_GM,*T*_2__ and *ϵ*_GM,*PD*_ are thresholds that can be tuned and optimized. The first two rules reflect the general appearance of the MS lesions. The rules that rely on Mahalanobis distance imply that only Gaussians that are not well explained by the healthy tissue models are suspected as lesion Gaussians. The last rule incorporates contextual information by reflecting our expectation to find lesions within the WM tissue. These rules are similar to rules used by Van Leemput et al. [19]. However, note that at this point decisions are made at the Gaussian level rather than the voxel level.

Clearly, a rule-based system is only one option for MS Gaussian detection. For example, a more supervised approach can be used: given examples marked by a human expert, standard classifiers can be applied to the Gaussian features. While we do not claim that the rule-based system suggested here is optimal (although it is intuitive and easy to implement), we do suggest that the detection on the Gaussian level provides greater strength in comparison to voxel-based detection.

Following the MS lesion Gaussian-detection stage, all the relabeled Gaussians now form a fourth class named MS lesion (MSL), with its own global intensity parameters. The EM is now applied to CGMM with four tissue types and segmentation of the tissues and the lesions is obtained as explained in Section 2. An illustration of the relabeling of the Gaussians can be seen in Figure 1(b).

## 4. The CGMM-CE Framework for Lesion Boundary Refinement

The segmentation induced from the CGMM yields good results in terms of lesion detection. However, there is usually an overestimation in the size of the lesions. This is most likely due to the fact that the area in the proximity of a lesion is somewhat different from the other tissues. In addition, the segmentation step of the CGMM model is done voxel-wise and does not take into account the smoothness of the lesion boundaries. Hence, a postprocessing step for refining the lesion boundary is needed. We use a modified version of the variational framework for segmentation of vector valued images, suggested by Rousson and Deriche [31] based on the Chan-Vese model [32]. In [31] a curve evolution equation for object-background image segmentation is derived based on statistical properties of the two regions. The intensity in each region is modeled using a Gaussian distribution. In our case, the lesion is the object and voxels around it are the background. The optimal lesion segmentation is defined by minimizing the energy:

(10) $$\begin{array}{c} F\left(\mu_{1},\Sigma_{1},\mu_{2},\Sigma_{2},\partial \Omega \right)=\overset{2}{\underset{i=1}{\sum }}\;\int_{\Omega_{i}}e_{i}\left(I\left(x\right)\right)+\text{length}\left(\partial \Omega \right), \end{array}$$

where Ω_1_ are the lesion region voxels, Ω_2_ are the nonlesion voxels, ∂Ω is the curve separating the two regions, *μ*_*i*_, Σ_*i*_ are the Gaussians parameters of Ω_*i*_ and *e*_*i*_(*I*) = −log*f*(*I* | *μ*_*i*_, Σ_*i*_). In level-set formulation, the functional from (10) takes a new form:

(11) $$\begin{array}{c} E\left(\Theta ,\phi \right)=\overset{2}{\underset{i=1}{\sum }}\;\int_{\Omega }\left[e_{i}\left(I\left(x\right)\right)\chi_{i}\left(\phi \left(x\right)\right)\right]dx+\text{length}\left(\partial \Omega \right), \end{array}$$

where *ϕ* is the level-set function, Θ is the parameter set {*μ*_1_, Σ_1_, *μ*_2_, Σ_2_}, and *χ*_1_, *χ*_2_ are the characteristic functions of regions 1 and 2, respectively: *χ*_1_(*ϕ*) = *H*_*ϵ*_(*ϕ*), *χ*_2_(*ϕ*) = 1 − *H*_*ϵ*_(*ϕ*) such that *H*_*ϵ*_(·) is a regularized Heaviside function. The length term is ∫_Ω_|∇*H*_*ϵ*_(*ϕ*(*x*))|*dx*. Note that the level-set formulation enables performing integration on the entire image domain, Ω. The energy (local) minimum can be obtained utilizing the level-set theory via a Gradient Descent (GD) method. From the GD equation, *ϕ*_*t*_ = −*δF*/*δϕ*, we obtain the following curve evolution equation:

(12) $$\phi_{t}\left(x\right)=\left(v\cdot \mathrm{div}\;\left(\frac{\nabla \phi }{\left|\nabla \phi \right|}\right)+e_{2}\left(I\left(x\right)\right)-e_{1}\left(I\left(x\right)\right)\right)\delta_{\epsilon }\left(\phi \left(x\right)\right).$$

**Figure 3:**
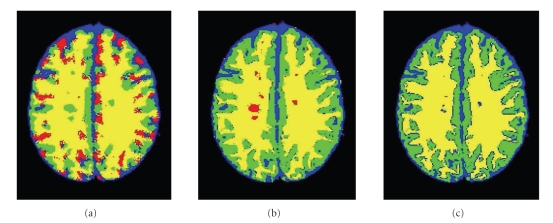
Illustration of MSL segmentation (BrainWeb data 9% noise, slice 105) using a four-phase curve-evolution (CE). Blue: CSF; Green: GM; Yellow: WM; Red: MSL. (a) Segmentation based a standard small-circles initialization followed by 30 iterations of four-phase curve evolution. (b) CGMM initial segmentation. (c) CGMM initial segmentation followed by five iterations of a four-phase curve-evolution.

Since brain images are complex, and since lesions are relatively small, it is impractical to use the standard initial contour (small circles) [31, 32] to find the three tissues and the lesions, simultaneously. The method is highly sensitive to the initial conditions for complicated four-phase images (such as brain images) as a gradient-descent optimization finds only local minima of the functional. Figure 3(a) shows a segmentation obtained from a four-phase curve-evolution using a standard circle-grid initialization [31]. It can be seen that (even with extensive manual tuning of the curve-evolution parameters) due to the complexity of the image and the added noise, we obtain poor brain segmentation results and no detection of MSL.

Using segmentation results of the three tissues and lesions obtained by CGMM just for initialization of a four-phase CE is not sufficient. Even with the improved CGMM initialization, the four-phase CE fails, as the image is too complex and the lesions are relatively small. Figure 3(c) shows an example of MSL segmentation results based on a CGMM segmentation followed by five iterations (that were needed until convergence) of four-phase curve evolution (the initial CGMM segmentation is shown in Figure 3(b)). As expected, and in agreement with Rousson and Deriche [31], the lesions gradually disappeared and the segmentation failed due to the complexity of the image.

In our approach we use a two-phase CE: lesions (object) versus nonlesion (background). We use the CGMM not just a starting point but also for background and object modeling. We model the lesion in the curve-evolution process using an intensity based Gaussian that is extracted from the lesion component of the CGMM model and is kept fixed during curve evolution iterations. We model the background using an intensity based GMM that is extracted from the CGMM model. Since there are three tissues that belong to Ω_2_, we define *e*_2_(*I*(*x*)) to be *min* (*e*_CSF_, *e*_GM_, *e*_WM_). This definition implies that the last two terms in the curve evolution equation are the log-likelihood ratio between MSL and the most likely normal-brain tissue. In [31] the energy is minimized with respect to both the Gaussian parameters and the boundary position. In our case, we avoid the parameter reestimation step. Instead, we use the global intensity parameters learned via the CGMM model. This enables us to continue enforcing the global constraints of the CGMM in the active contour step. For example, if one of the slices has an initial contour that encircles only false positives, then the constraint will usually cause it to die off, whereas parameter estimation during the curve evolution is likely to cause the system to learn the parameters of the false lesion. To summarize, three modifications are suggested in the current work, in applying the Rousson-Deriche framework to the MSL delineation task.

- The CGMM lesion segmentation is used as the initial contour.
- The background is modeled by a GMM (CSF, GM, WM) instead of a single Gaussian.
- Parameter estimation during the curve evolution process is avoided; CGMM intensity parameters are used instead.

The lesion detection obtained using CGMM, therefore, makes it possible to apply active contour techniques for lesion delineation.

We dub the proposed method, which is based on CGMM followed by a curve evolution refinement, CGMM-CE. The steps of the CGMM-CE algorithm that were presented in Sections 2, 3, and 4 are summarized below.

- Apply intensity based K-means to obtain an initial clustering of the voxels into three tissues.
- Form the initial spatial Gaussians based on clustering of the connected components of each tissue. Set the Gaussian labels according to the tissue they belonged to.
- Utilize fast KDT to compute the nearest Gaussians for each voxel.
- Apply the EM algorithm to find the probabilistic model for the three tissues.
- Find the lesion Gaussian using Gaussian outlier detection.
- Apply the EM algorithm to find the probabilistic model for the four “tissues” (three healthy tissues + lesions).
- Find lesion segmentation based on the probabilistic model.
- Utilize curve evolution techniques to refine the lesion boundaries.

## 5. Experimental Results

Lesion segmentation validation is a challenging task. First, with notable exceptions there are hardly any data sets that are publicly available for the research community. Second, there is no clear convention for the best measures to quantify the results. Third, the nature of the lesions is almost never binary. It is a challenge to decide where the lesion ends and the healthy tissue starts, a fact that introduces variability in human-expert markings. Manual segmentation of large data sets is a labor and time-consuming task. This makes it difficult to produce ground truth segmentation for large data sets. In the current work, we demonstrate results on a the Brainweb online database [33] which is considered to be a standard benchmark upon which MRI brain image segmentation algorithms are tested and compared. An initial result on a real brain, is shown as well. 

### 5.1. BrainWeb Simulated MRI Data

The CGMM-CE algorithm was tested on bias-free simulated MRI data taken from the BrainWeb online database [33]. The following coregistered modalities were used: *T*_1_, *T*_2_ and *PD*. Voxel size was 1 mm^3^. Experiments were done on 61 slices (axial slices 60–120) that contain 93% of the lesion burden. Different levels of noise were added: 3%, 5%, 7% and 9%. According to BrainWeb's web-site, the noise in the simulated images has Rayleigh statistics in the background and Rician statistics in the signal regions. The “percent noise” number (e.g., 5% noise) represents the percent ratio of the standard deviation of the additive white Gaussian noise versus the signal for a reference tissue. In the brain images under observation the noise reference tissues were WM for *T*_1_, and CSF for *T*_2_ and *PD*.

**Figure 4:**
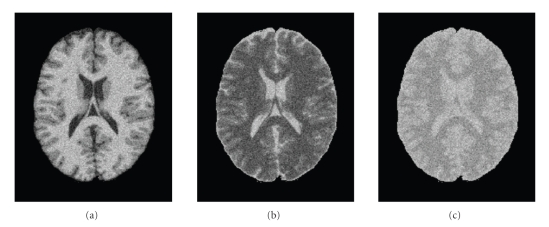
BrainWeb data with 9% noise level (a) *T*_1_, (b) *T*_2_, (c) *Pd*.

Figure 4 shows slice 90 with different levels of noise. Visual inspection suggests that images with 9% noise present a challenge even for manual segmentation. We are aware that the noise level in real data is almost always less than 9%. We provide the 9% segmentation result to demonstrate the robustness of the CGMM-CE method.

**Figure 5:**
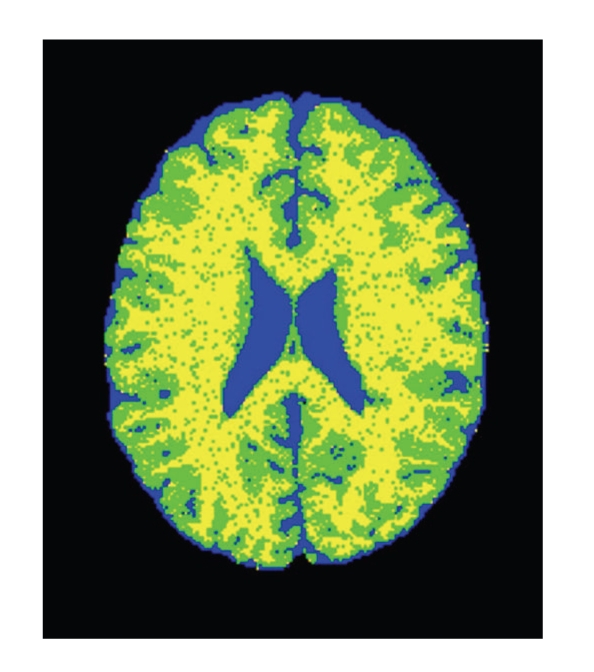
Intensity K-means segmentation. *K* = 3. BrainWeb data, slice 95 with 9% noise. Blue: CSF; Green: GM; Yellow: WM.

As preprocessing steps, the brain region was extracted and the intensity distribution of each channel was normalized to have zero mean and unit variance. Then, a K-means intensity-based clustering was performed with *K* = 3, to achieve an initial crude segmentation (see Figure 5). The global intensity parameters of each tissue were initialized as the sample mean and sample covariance of the extracted tissue segment.

Following the CGMM parameter initialization and KDT computation (with 10 neighbors), a subset of the Gaussians was relabeled as MSL. For every Gaussian, a set of decision rules was applied. If all the conditions described in this set held, and in addition the majority of Gaussian's 10 nearest Gaussians were labeled as WM, then the Gaussian was relabeled as MSL. The selected subset formed a fourth class. Optimal MSL parameters were found using the EM algorithm. In all the experiments, it took less than seven iterations to reach convergence. Finally, the curve evolution step was applied to refine the lesion segmentation. The velocity, *v*, in (12), was chosen as a constant: *v*(*x*, *y*) = const. The constant value was set to be 5, 3, 1, 1 for noise levels 3, 5, 7, 9, respectively (higher velocity for low noise levels).

Performance is evaluated via a comparison with Van Leemput's state-of-the-art algorithm (hereon termed KVL) [19] implemented by the EMS software packagehttp (http://www.medicalimagecomputing.com/EMS). In the KVL implementation, the statistical brain atlas of the SPM99 (http://www.fil.ion.ucl.ac.uk/spm/) was normalized to the target brain volume images. Note that KVL is the only one who actually made his code/toolbox available, so the comparison is fair. Usually people report on lesion detection on MRI images with 3% noise level. We have not found reported results on 9% (or in fact, even 7%) other than KVL [19].

**Figure 6:**
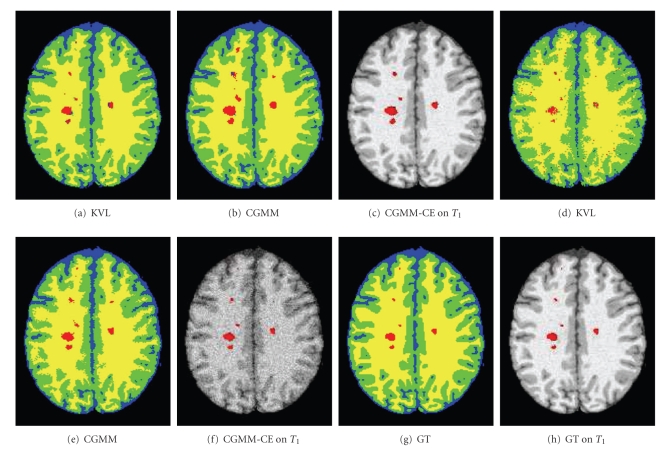
Segmentation results on BrainWeb data, slice 105. Blue: CSF; Green: GM; Yellow: WM; Red: MSL. (a)–(c) 3% noise, (d)–(f) 9% noise, (g) and (h) ground truth.

Figure 6 shows segmentation results on a single slice. Results are shown for both KVL, CGMM, and CGMM-CE methods. Figures 6(a)–6(c) shows a low noise case of 3%, Figures 6(d)–6(f)  shows a high noise case of 9%, and Figures 6(g)  and 6(h)  show the ground truth MS lesion segmentation. Visual inspection indicates that for a low level of noise (3%), both algorithms present results that are close to the ground truth. For strong noise scenarios (9%), the CGMM produces a better and smoother healthy tissue segmentation. Also note that in terms of detection of whole MS lesions (e.g., number of false positives), the CGMM presents more successful results. Following the curve evolution step, the CGMM-CE framework presents further improved results.

**Figure 7:**
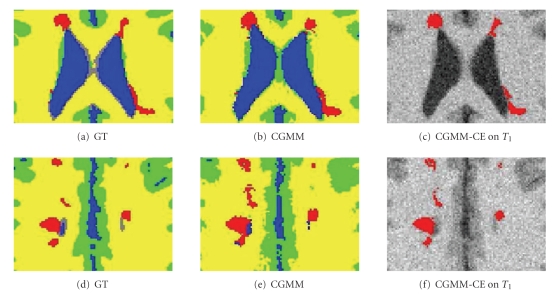
Zoom in of segmentation results. Blue: CSF; Green: GM; Yellow: WM; Red: MSL. Upper row: slice 97. Lower row: slice 104.

The need for boundary refinement following the CGMM segmentation, and the result of applying the curve evolution process are shown as a zoom-in in Figure 7. Two slice examples are shown including for each is the ground-truth (left), the CGMM segmentation result (center), and the refined CGMM-CE segmentation (right). In the bottom left of Figure 7(e) we can see that two distinct lesions were joined together in the CGMM segmentation. Figure 7(f) shows that the refined contour succeeded in separating these two lesions. Comparing these two images further, we can also see that the overestimation of several of the lesions has significantly decreased. Similar results have been observed in other such slices examined. Note that in Figure 7(c) the refined lesion split to two very close adjacent lesions, this is not a general feature of the CE, as the fact that the CGMM (see Figure 7(b)) produced a lesions whose two parts are almost disconnected. The reason for this is that only three main healthy tissues are modeled, but in fact the data presents about 9 or 10 healthy tissues, most of them are typically ignored. This particular lesion is on top such a tissue—the Glilal matter and therefore few pixels are missed, which resulted in a final insignificant splitting.

A quantitative comparison between the algorithms was made using the Dice overlap metric which measures the overlap between the automatic segmentation and the ground truth for each tissue *j*. This performance index is often used in assessment of medical image segmentation algorithms, and is given by

(13) $$\begin{array}{c} \text{Dice}\;\text{Index}=\frac{2V_{ae}^{j}}{V_{a}^{j}+V_{e}^{j}}, \end{array}$$

where *V*_*as*_^*j*^ is the number of voxels that are assigned to tissue *j* by both the ground truth and the automated algorithm, *V*_*a*_^*j*^ and *V*_*e*_^*j*^ denote the number of voxels assigned to tissue *j* by the algorithm and the ground truth, respectively. Dice values vary between zero (no agreement with the ground truth segmentation) and one (perfect agreement with ground truth segmentation).

Table 1 shows MS lesion Dice results for KVL and the CGMM-CE methods. In the 3% case, KVL is slightly better. For higher values of noise, the performance improvement of the CGMM-CE framework over the KVL is notable. It should be noted that for lesion segmentation, Dice values above 0.70 are considered to be clinically stratifying [16]. Moreover, a comparison between two different segmentations given by two different human experts, is likely to yield Dice index lower than 0.70 [18].

The computation time (using Pentium (R) 4 CPU 3.40 GHz, 1.49 GB of RAM) for each significant part of the algorithm is initialization: 3 minutes, KDT: 2 minutes, MS Gaussian detection: 1 minute, and EM iteration: 1 minute. The curve evolution computation time was insignificant. This is partially due to the fact that the CGMM provided a starting point that is very close to the local minima of the functional and thus the number of curve evolution iterations was usually small. Also, since we used the CGMM intensity parameters, there was no need to reestimate these parameters for every iteration. Thus, the entire CGMM-CE process takes less than a quarter of an hour for BrainWeb data, slices 60–120.

### 5.2. Results on Real Data

**Figure 8:**
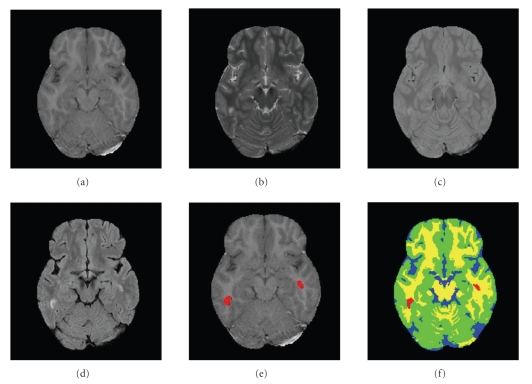
Real brain images, example 1. (a) *T*_1_ (b) *T*_2_ (c) *PD* (d) *FF* (e) Manual segmentation of MS lesions (red) overlayed on *T*_1_ (f) CGMM-CE segmentation. Blue: CSF; Green: GM; Yellow: WM; Red: MSL.

**Figure 9:**
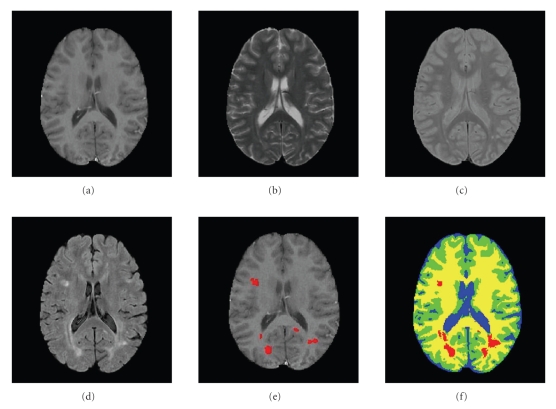
Real brain images, example 2. (a) *T*_1_, (b) *T*_2_, (c) *PD*, (d) *FF*, (e) Manual segmentation of MS lesions (red) overlayed on *T*_1_, (f) CGMM-CE segmentation. Blue: CSF; Green: GM; Yellow: WM; Red: MSL.

In what is still a work in progress, the algorithm was also tested on real MRI data. These experiments were performed in a joint effort with the MS Center at Sheba Medical Center, Israel. Here, ground truth segmentation of the healthy tissues is not available. However, we have manual MS lesion segmentation, provided by a human expert. In these experiments, the Fast Flair (FF) modality, was added to *T*_1_, *T*_2_, and *PD* which were previously used. Note that the BrainWeb data [33] does not offer this modality. The FF provides a good contrast between CSF and the other healthy tissues (GM and WM). To some extent, it also provides a good contrast between MS lesions and the three healthy tissues. The main purpose of FF in MS is to suppress false positive from CSF signals. Preprocessing steps include conventional procedures such as extraction of the brain region (also know as skull removal); coregistration of the images from the different channels; and bias filed correction. Once the preprocessing is finalized, we extract from each voxel both intensity and spatial features. Unlike the isotropic voxel used in the BrainWeb data experiment, here the voxel size was 1 mm × 1 mm × 3 mm. The set of decision rules for Gaussian detection was similar to the one used in the BrainWeb dataset, with the addition of rules that take the FF into account (e.g., lesions are brighter than all the other tissues). Results on real data are shown in Figures 8  and 9. In both examples it is possible to see a smooth and visually plausible segmentation of the healthy tissues. With regard to the lesion detection and delineation, the main lesions are in fact detected (except for one in Figure 9).

## 6. Discussion

 In [26], the CGMM was shown to be a successful framework for healthy tissue segmentation of *T*_1_ MRI brain images. In the current work, the CGMM-CE framework was presented, as a methodology to detect and delineate MS lesions within multichannel input data. A novel model approximation scheme has been suggested to accelerate computing time by several orders of magnitude. Finally, a curve evolution technique was applied to refine the segmentation of the MS lesions. CGMM was shown here to provide a successful parametric framework for healthy tissue and MS lesion segmentation in abnormal brains, without requiring prior registration to a brain atlas. Unlike most MS lesion segmentation algorithms, here detection of MS lesions is done on the Gaussian-level rather than the voxel-level. Besides the fact that it ensures smoother segmentation and an inherent robustness to noise, Gaussian-level reasoning incorporates spatial information in a simple and intuitive way (unlike MRF that is often used in the literature). 

One of the advantages of the suggested rule-based system for MS Gaussian detection, in addition to its simplicity, is that it enables the user (the radiologist) to tune the parameters in a clear and understandable way. Also, users can easily add or remove rules to match the rules that they himself apply for manual lesion segmentation. For example, they can decide to exclude all Gaussians that are close to the centerline of the brain. While similar rules were used in the literature, here the rules are applied to Gaussians (that correspond to small regions) rather than to voxels. Of course, the rule-based system for MS Gaussian detection is only one option for classifying Gaussians. For example, it is possible to use the markings given by a human expert to train classifiers such as a support vector machine.

The Gaussian KDT enables a fast implementation of the CGMM framework and provides additional contextual information. The KDT can be useful for other GMM applications in which the number of Gaussians is large. The curve evolution technique suggested by Rousson and Deriche [31] was utilized to refine the CGMM lesion segmentation. Since brain images are complex, this technique cannot be directly applied to the MRI data. The CGMM segmentation provides an excellent initial contour, so the Gradient-Descent limitations are reduced. The use of the optimal MSL intensity parameters from the CGMM, eliminates the need to reestimate them in each curve evolution, thus speeding the process. In experiments with simulated data, both visual and quantitative comparisons with the KVL algorithm have demonstrated better results, especially as the noise increases. Typical results of MSL segmentation reported in the literature refer to 3% noise only (e.g., the sate-of-the-art algorithm suggested by Akselrod-Ballin et al. [15]). The proposed algorithm performs well with increased noise up to 9%. Experiments with real MRI data yielded promising results. We are currently extending the experiments to additional real brain datasets, in a joint effort with the MS center at Sheba hospital. Also planned is the validation across multiexpert ground-truth data. Some additional work needs to be done to address the very small size lesions, and to achieve more accurate delineation. Multiple-expert markings need to be compared as well, in order to estimate the observer variability on this data set, which can then be compared to the automated algorithm performance.

## Figures and Tables

**Table 1:** Dice index for lesion segmentation on BrainWeb data for different noise levels.

Algorithm	Noise			
	3%	5%	7%	9%
KVL	0.80	0.73	0.61	0.47
CGMM+CE	0.79	0.79	0.78	0.76
